# Understanding the underlying genetic mechanisms for age at first calving, inter-calving period and scrotal circumference in Bonsmara cattle

**DOI:** 10.1186/s12864-023-09518-8

**Published:** 2023-08-24

**Authors:** Jason J Reding, Robert R van der Westhuizen, Donagh P Berry, Este van Marle-Köster

**Affiliations:** 1 Department of Animal Sciences, University of Pretoria, Hatfield, 0028 South Africa; 2 Teagasc - The Irish Agriculture and Food Development Authority, Moorepark, Fermoy, Cork Ireland

**Keywords:** Bioinformatics, Deregression, Estimated breeding values, Genomics, Regression, Single nucleotide polymorphism

## Abstract

**Background:**

Reproduction is a key feature of the sustainability of a species and thus represents an important component in livestock genetic improvement programs. Most reproductive traits are lowly heritable. In order to gain a better understanding of the underlying genetic basis of these traits, a genome-wide association was conducted for age at first calving (AFC), first inter-calving period (ICP) and scrotal circumference (SC) within the South African Bonsmara breed. Phenotypes and genotypes (120,692 single nucleotide polymorphisms (SNPs) post editing) were available on 7,128 South African Bonsmara cattle; the association analyses were undertaken using linear mixed models.

**Results:**

Genomic restricted maximum likelihood analysis of the 7,128 SA Bonsmara cattle yielded genomic heritability’s of 0.183 (SE = 0.021) for AFC, 0.207 (SE = 0.022) for ICP and 0.209 (SE = 0.019) for SC. A total of 16, 23 and 51 suggestive (*P* ≤ 4 × 10^-6^) SNPs were associated with AFC, ICP and SC, while 11, 11 and 44 significant (*P* ≤ 4 × 10^-7^) SNPs were associated with AFC, ICP and SC respectively. A total of 11 quantitative trait loci (QTL) and 11 candidate genes were co-located with these associated SNPs for AFC, with 10 QTL harbouring 11 candidate genes for ICP and 41 QTL containing 40 candidate genes for SC. The QTL identified were close to genes previously associated with carcass, fertility, growth and milk-related traits. The biological pathways influenced by these genes include carbohydrate catabolic processes, cellular development, iron homeostasis, lipid metabolism and storage, immune response, ovarian follicle development and the regulation of DNA transcription and RNA translation.

**Conclusions:**

This was the first attempt to study the underlying polymorphisms associated with reproduction in South African beef cattle. Genes previously reported in cattle breeds for numerous traits bar AFC, ICP or SC were detected in this study. Over 20 different genes have not been previously reported in beef cattle populations and may have been associated due to the unique genetic composite background of the SA Bonsmara breed.

## Background

The improvement of reproductive efficiency is of major economic importance in beef production systems and improvements in total lifetime productivity is a key metric for efficiency, which is a function of both reproduction and total output per cow [1, 2]. In South Africa (SA), the majority of beef is produced under extensive production systems with average calving percentages (i.e., the proportion of cows that give birth in comparison to the number of cows that could possibly give birth) of 62% [3]. Bonsmara cattle are a beef breed, developed during the nineteen-sixties, resulting in a composite comprised of approximately 5/8 Afrikaner and 3/8 exotic breeds [4]. It is the largest breed represented in the seed stock and commercial beef industry in SA. As the beef industry primarily relies on reproductive efficiency, a reduction in the unproductive period of a female’s life would positively impact production costs and profit as well as having a favourable impact on the carbon footprint.

Genetic improvement in reproductive performance remains challenging, hindered by the generally low associated heritability (h^2^), coupled with the complexity of recording these traits and/or the late expression of reproductive traits [5–7]. The age at onset of puberty can impact overall productivity through the heifer becoming productive at an earlier age [8]. Puberty is experienced earlier in composite and crossbred animals compared to their purebred counterparts with the same trend observed for early maturing versus late maturing beef breeds [9]. Age of puberty is however difficult to record, and therefore age at first calving (AFC) is often used as an indicator of heifer fertility. The heritability estimates reported for AFC differ depending on the breed, with [10] estimating a h^2^ = 0.10 in Brahman cattle, [8, 11, 12] reporting on Nellore cattle (0.01 to 0.31) and [13] estimating a h^2^ = 0.08 for AFC in Angus and Hereford cattle. Recent studies on South African populations are limited with [14] reporting a h^2^ = 0.08 in Bonsmara cross cattle, while [5] reported higher heritability for AFC in Afrikaner (0.27) and Drakensberger (0.30) Sanga cattle breeds. Inter-calving period (ICP) is a relatively easy trait to record and is included in most beef and dairy breed societies recording schemes in South Africa [15]. Estimates of ICP yield low to moderate heritability (0.01–0.10) in Brahman and composite beef cattle breeds [6, 10, 16]. As scrotal circumference (SC) in bulls is relatively easy to measure, with a moderate to high heritability [6, 12, 17–19], it has been suggested as an indicator trait for age at puberty, the latter being resource-intensive to measure.

Several studies have reported positive genetic correlations between SC and growth traits like mature body weight (r_g_ = 0.37–0.40) in composite beef cattle [20], weaning and mature weight (r_g_ = 0.60–0.72) in *Bos indicus* cattle [21] as well as weaning (r_g_ = 0.312) and yearling (r_g_ = 0.519) weight in Nellore cattle [22]. Although weaker genetic correlations between SC and weaning weight (r_g_ = 0.15) have been reported in SA Bonsmara bulls [23], genomic analyses of SC and SC-related traits have identified genes which are also known to associate with growth traits [24].

Genome-wide association studies (GWAS) conducted on European and tropically adapted beef cattle breeds revealed several potential genes for fertility traits including AFC [8, 12, 25, 26], ICP [27], pregnancy status [28], gestation length [29], sexual precocity [30] and SC [12, 31, 32]. Some studies have combined fertility traits with body weight at puberty [33] while multi-trait meta-analyses have also been applied [12], which indicated that similar regions of the genome harbour genetic variation that potentially influence reproductive traits in both genders.

The SA Bonsmara, classified as a Sanga type, is a unique composite breed of 3/8 exotic (Milk Shorthorn, Hereford) and 5/8 Afrikaner [4]. The breed was established through a well-documented crossbreeding program, with the aim of founding a local composite breed that was well adapted to the challenges of a diverse SA climate. This study was the first attempt to apply GWAS for fertility traits in SA Bonsmara cattle to provide insight on gene regions in the SA Bonsmara. The objective of this study was to perform a genome-wide association study for three reproductive traits (AFC, ICP and SC) in a South African Bonsmara population in order to identify quantitative trait loci (QTL) for these traits.

## Results

### Variance components estimation

A genetic correlation of 0.37 was estimated between AFC and ICP in the SA Bonsmara using the breeding values derived from the bivariate model. Pedigree heritability estimates were 0.22 for AFC, 0.13 for ICP and 0.38 for SC. The genomic REML analysis yielded genomic heritability and standard errors (SE) of 0.183 (SE = 0.021) for AFC, 0.207 (SE = 0.022) for ICP and 0.209 (SE = 0.019) for SC.

### Genomic population quality control

**Fig. 1:**
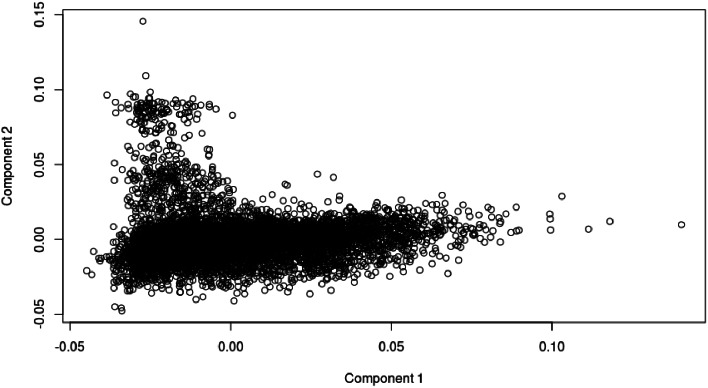
Multidimensional Scaling of 7,231 SA Bonsmara Genotypes

**Fig. 2:**
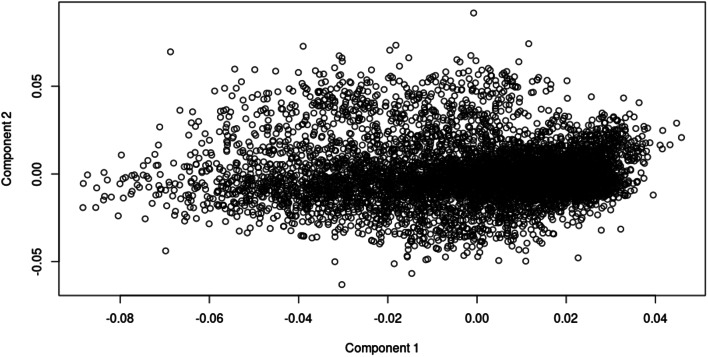
Multidimensional Scaling of 7,128 SA Bonsmara Genotypes

Individual based quality control resulted in the omission of ninety-five SA Bonsmara genotypes across the five genotyping arrays available. Assessment of identical by state (IBS) genetic distances yielded a multidimensional scaled plot (MDS; Fig. 1). Identification of outliers as well as their genotyped progeny led to the further removal of 103 genotypes (Fig. 2) resulting in a final sample population of 7,128 SA Bonsmara animals (4,403 males, 2,725 females). The effect of filtering out animals with reliabilities smaller than 0.01 and an effective record count (ERC) of less than 0.50 culminated in genome-wide population sizes of 4,460 animals for AFC (median ERC = 1.758), 4,276 animals (median ERC = 1.717) for ICP1, and 5,452 animals for SC (median ERC = 1.004).

### Age at first calving

**Fig. 3:**
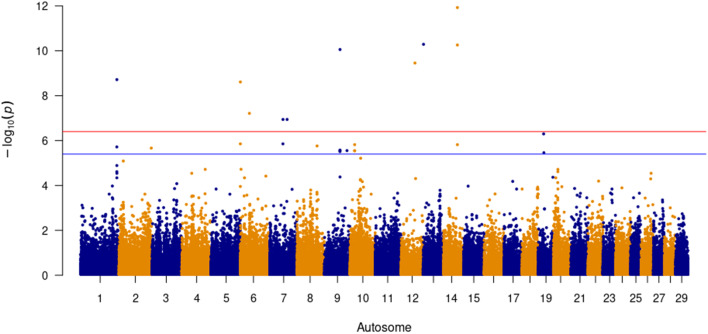
Significant SNP above the red line (≤ 4 × 10^− 7^) for Age at First Calving (4,460 animals)

Eleven single nucleotide polymorphisms (SNPs) at the genome wide threshold p-value of less than 4 × 10^− 7^ and a further 16 suggestive SNPs at a p-value of less than 4 × 10^− 6^ were associated with AFC (Fig. 3). Pair-wise linkage disequilibrium (LD) analysis of significant SNPs resulted in the identification of 11 QTL across eight autosomes (Table 1). A total of 11 different genes were co-located with the associated QTL, with the frequency of the major alleles being between 0.527 and 0.982, respectively. *Bos taurus* autosome (BTA) 7 harboured two QTL containing nine genes (*ARAP3*, *CLINT1*, *FCHSD1*, *LSM11*, *PCDHGA*, *PCDHGB*, *PCDHGC*, *RELL2* and *THG1L*) with each of these QTL having seven SNPs in pair-wise LD. The most significant QTL, located on BTA 17, was a single intergenic SNP with a minor allele frequency (MAF) of 0.13. The gene, *PLCB1*, resides in a QTL that spans four SNPs in pair-wise LD (r^2^ > 0.50), 212.17 kilobase pairs (kbp) across BTA 13, with the significant SNP (BovineHD1300000449; MAF = 0.15), associating with AFC.

**Table 1:** List of identified QTLs and genes associated with Age at First Calving

BTA^1^	SNPs in QTL^2^	Start - End	Significant SNP	P-value	MAF^3^	Genes in quantitative trait loci
1	1	149305906–149305906	BTB-00069838	1.92E-09	0.070	
6	6	260882–524856	BTA-94560-no-rs	2.45E-09	0.392	*ENSBTAG00000032764*
6	6	37840605–37850593	BovineHD0600010469	6.13E-08	0.326	
7	7	54096162–54433589	ARS-BFGL-NGS-11368	1.15E-07	0.132	*ARAP3, FCHSD1, PCDHGA, PCDHGB, PCDHGC, RELL2*
7	7	71366963–71525148	BTA-79723-no-rs	1.15E-07	0.224	*CLINT1, LSM11, THG1L*
9	1	64310181–64310181	BovineHD0900017655	8.78E-11	0.240	
12	3	57271355–57364937	BTB-01886351	3.50E-10	0.448	*CNV*
13	4	1655502–1867669	BovineHD1300000449	5.13E-11	0.146	*PLCB1, CNV*
14	1	58874718–58874718	BovineHD1400016348	5.51E-11	0.330	
14	3	58902500–58967114	BovineHD1400016360	1.18E-12	0.473	
17	1	19384978–19384978	BTA-16045-no-rs	8.90E-16	0.127	

^1^*BTA Bos taurus* autosome

^2^*SNPs in QTL* Number of single nucleotide polymorphisms in this quantitative trait loci

^3^MAF Minor allele frequency of the significant single nucleotide

### Inter-calving period

**Fig. 4:**
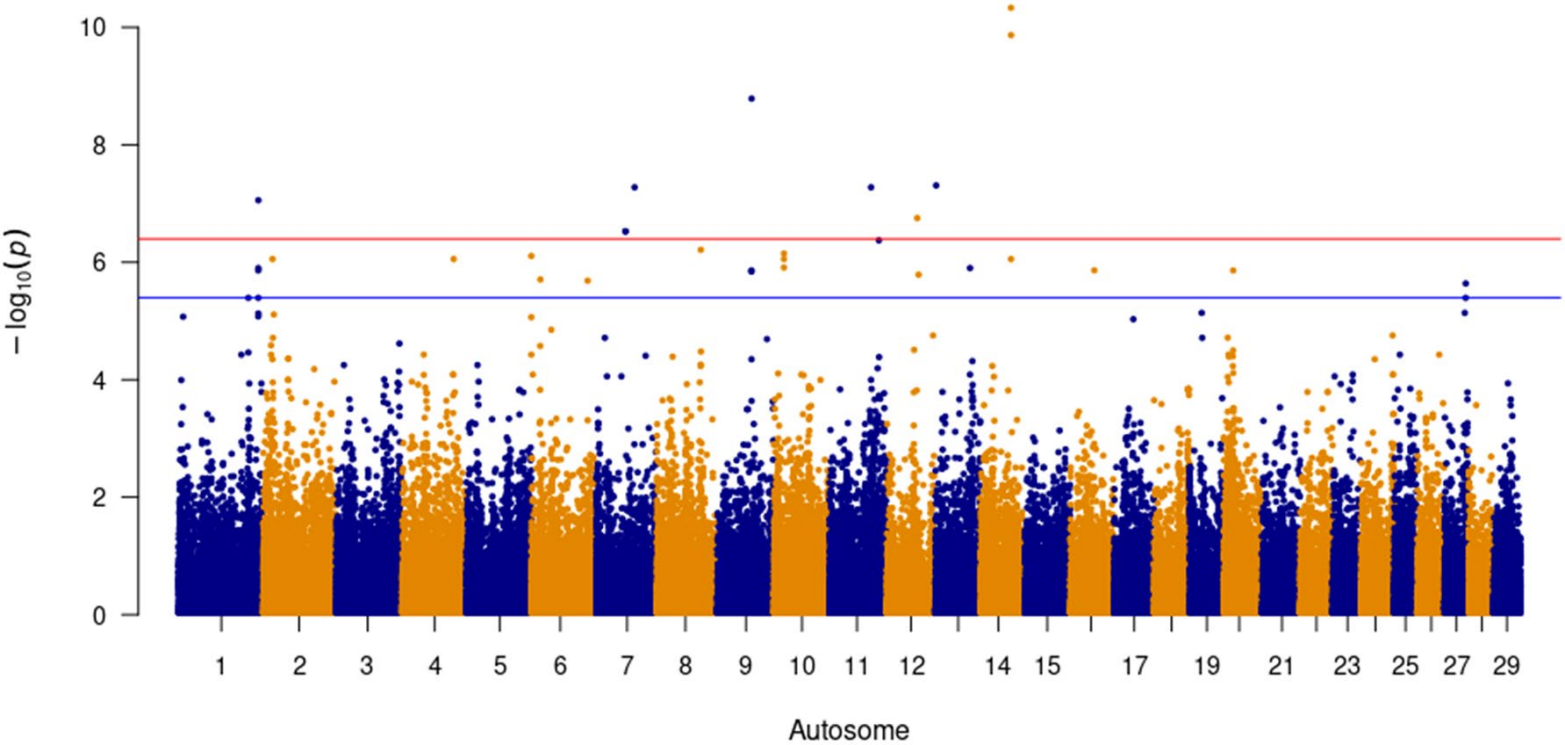
Significant SNP above the red line (≤ 4 × 10^− 7^) for Inter-Calving Period (4,276 animals)

Eleven SNPs were significantly associated with ICP, with a further 23 SNPs being suggestively associated (Fig. 4). The most significant SNP (BTA-16,045-no-rs; *P* = 2.83 × 10^− 16^, MAF = 0.13) was on BTA 17 and was an intergenic SNP. A total of ten QTL across eight autosomes were significantly associated with ICP (Table 2). A QTL 337.43 kbp in length containing six genes (*ARAP3*, *FCHSD1*, *PCDHGA*, *PCDHGB*, *PCDHGC* and *RELL2*) and a second QTL of 158.19 kbp in length harbouring three genes (*CLINT1*, *LSM11* and *THG1L*) were both positioned on BTA 7. A QTL containing the *LDAH* gene, is a 131.85 kbp long and contains two SNPs in high LD (r^2^ = 0.74).

**Table 2:** List of identified QTLs and genes associated with Inter Calving Period

BTA^1^	SNPs in QTL^2^	Start - End	Significant SNP(s)	P-value	MAF^3^	Genes in quantitative trait loci
1	1	149305906–149305906	BTB-00069838	8.79E-08	0.069	
7	7	54096162–54433589	BovineHD0700015625	2.98E-07	0.129	*ARAP3, FCHSD1, RELL2, PCDHG(A/B/C)*
7	7	71366963–71525148	BTA-79723-no-rs	5.30E-08	0.223	*CLINT1, THG1L, LSM11*
9	1	64310181–64310181	BovineHD0900017655	1.63E-09	0.235	*CNV*
11	2	78114859–78246711	ARS-BFGL-NGS-16050	5.30E-08	0.325	*LDAH*
12	3	57271355–57364937	BTB-01886351	1.77E-07	0.444	*CNV*
13	4	1655502–1867669	BovineHD1300000449	4.92E-08	0.146	*PLCB1, CNV*
14	1	58874718–58874718	BovineHD1400016348	4.65E-11	0.333	
14	3	58902500–58967114	BovineHD1400016360	1.36E-10	0.477	
17	1	19384978–19384978	BTA-16045-no-rs	2.83E-16	0.129	

^1^BTA Bos taurus autosome

^2^SNPs in QTL Number of single nucleotide polymorphisms in this quantitative trait loci

^3^MAF Minor allele frequency of the significant single nucleotide

### Scrotal circumference

**Fig. 5:**
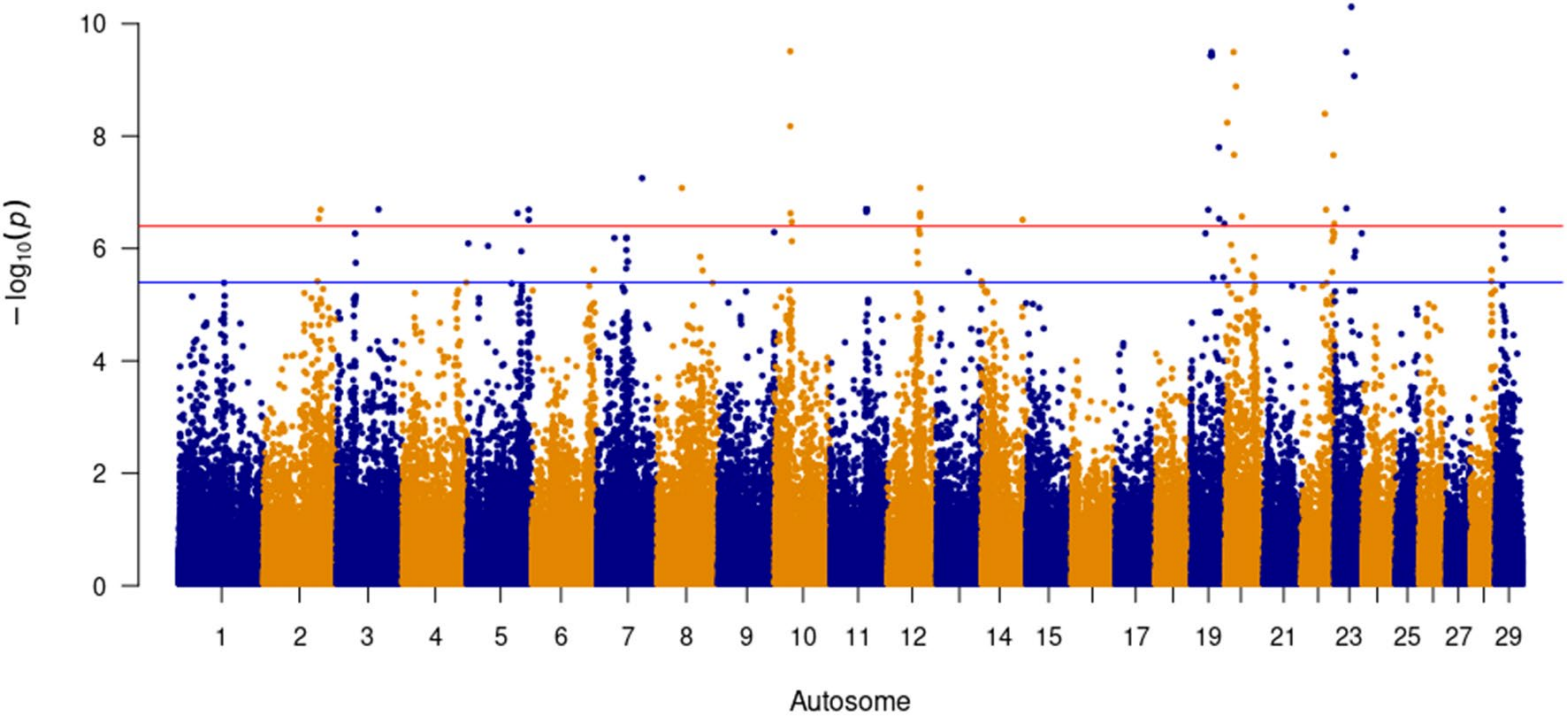
Significant SNP above the red line (≤ 4 × 10^− 7^) for Scrotal Circumference (5,452 animals)

Forty-four SNPs were significantly (*P* ≤ 4 × 10^− 7^) associated with SC with a further 51 SNPs being suggestive (*P* ≤ 4 × 10^− 6^, Fig. 5). Pair-wise LD analysis identified a total of 41 QTL across 14 autosomes (Table 3). The most significant SNP, (BovineHD2300009170; *P* = 5.02 × 10^− 11^, MAF = 0.04), which is lowly segregating in the SA Bonsmara population, was an intron variant of *SLC17A3* located on BTA 23. A QTL consisting of eleven SNPs in LD (r^2^ > 0.50) on BTA 11 consisted of downstream variants for the gene *NEURL1B*. Gene rich QTL were located on BTA 2, 11, 19 and 22. A 93.2 kbp QTL on BTA 2 consisting of six SNPs harboured the gene *ABCA12*, a small nucleolar RNA (*RF00156*) and an insertion/deletion copy number variant (CNV). Five QTL spanning a 1.65 Mega basepair (Mbp) portion of BTA 11 house the genes *AAK1*, *ANTXR1*, *CNRIP1*, *GMCL1*, *MXD1*, *PP3R1* and *SNRNP27*. A homeobox gene dense QTL on BTA 19 sweeping 171.78 kbp consisted of four *HOXB* gene variants and *TTLL6*. On BTA 22, a significant SNP (Hapmap33950-BES3_Contig483_1359; *P* = 4.02 × 10^− 9^, MAF = 0.425) in moderate LD with two flanking SNPs (downstream r^2^ = 0.524, upstream r^2^ = 0.527) includes the genes *ABHD6*, *PXK* and *RPP14* associated with SC in this study. The QTL with the most genes, namely *ALDH1L1*, *CHST13*, *C22H3orf22*, *SLC41A3*, *TXNRD3*, *UROC1* and *ZXDC*, was comprised of five SNPs over a 137.92 kbp DNA region on BTA 22.

### Overlapping genes across traits

In this study, three QTL, two on BTA 7 and one on BTA 13, were detected to be associated with more than one reproductive trait. The QTL on BTA 13 contains four SNPs in LD, with the SNP (BovineHD1300000449) being significantly associated with both AFC (*P* = 5.13 × 10^–11^) and ICP (*P* = 5.13 × 10^− 8^). Two large aforementioned QTL located on BTA 7 are both significantly associated with AFC and ICP. The first QTL was significantly associated with both AFC (*P* = 1.15 × 10^− 7^) and ICP (*P* = 2.98 × 10^− 7^), while the SNP (ARS-BFGL-NGS-11368), located in this same QTL was significantly associated with ICP (*P* = 2.98 × 10^− 7^). The second QTL, with seven SNPs in LD with each other, was identified to be significantly linked to both AFC (*P* = 1.15 × 10^− 7^) and ICP (*P* = 5.30 × 10^− 8^).

## Discussion

This study was the first attempt to gain insight into the underlying genetic mechanisms for reproductive traits in a South African beef cattle breed. The pedigree and genomic based heritability estimates for AFC, ICP and SC were similar to those identified in studies in beef cattle breeds [6, 8, 10–14, 17–19]. The low to moderate heritability estimates for most reproductive traits and limited genomic studies on indigenous breeds in Southern Africa [34–36] justifies the further investigation of this study. Genomic tools hold the potential to unlock invaluable information [37], that may help explain physiological conditions that currently remain unanswered [38].

### Age at first calving

In this study, a total of eleven significant SNPs associated with AFC were observed; none of these have been previously reported in *Bos indicus* and crossbred beef cattle to be associated with AFC. Of the eleven genes and two CNVs located in QTL associated with AFC, ten of these genes have not been previously associated with AFC or sexual precocity in beef cattle populations but have been reported in human [39] and sheep [40] populations. Four of the genes observed in this study (*ENSBTAG00000032764*, *FCHSD1*, *PLCB1* and *RELL2*) have been previously associated in beef cattle populations [41–43] with two of these genes involved in more than one trait. Physical growth and body composition have a developmental effect on the reproductive organs which would determine the onset of puberty and subsequent AFC [44, 45]. *ENSBTAG00000032764* (pseudogene) was previously reported as a candidate gene associated with carcass traits [41]. The resultant translated protein facilitates the storage of iron in a soluble, non-toxic form, which is an essential component for iron homeostasis.

### Inter-calving period

The inter calving period which is the period between two calvingss has the lowest genetic and genomic heritability of all three traits in this study and, in practice, is a function of the ability to ovulate post-calving [45], express oestrus, establish and maintain pregnancy and gestation length [29, 46]. A total of eleven genes and three CNVs were based in QTL associated with ICP which included ten genes not previously associated with ICP or reproduction in beef cattle. The 337.43 kbp QTL on BTA 7 contains three genes (*PCDHGA*, *PCDHGB*, *PCDHGC*) from the protocadherin (*PCDH*) family group. Studies have shown the *PCDH* genes play an integral role in ovarian follicular [47] and embryonic [48] development. *LDAH* is a lipid droplet-associated hydrolase that is essential in lipid storage and was previously associated with feet and leg disorders in Danish Holstein cattle [49].

### Scrotal circumference

Pair-wise LD analysis identified a total of 41 QTL across 14 autosomes (Table 3). Genes not previously reported in beef cattle populations include *AAK1*, *ACAD19*, *CHST13*, *CNRIP1*, *CRISP1*, *DERA*, *KIF2A*, *PARVB*, *PNPO*, *PXK*, *RPP14*, *SKAP1*, *SLC41A3*, *SNRNP27*, *SSBP2*, *TCOM5B*, *TJP2*, *TTLL6*, *UBE2Z*, *UROC1* and *ZXDC*. Most of these genes have not been previously reported to be associated with SC or sperm-related traits, but upon further investigation into the biological pathways that the genes are involved in, it becomes clear that the genes may play some role in the expression of fertility related traits. Biological pathways discussed below include carbohydrate catabolic processes (*CHST13*, *DERA* and *UROC1*), cellular development (*HOXB5*, *HOXB7*, *HOXB9*, *HOXB13* and *TTL13*), lipid metabolism (*ABCA12*, *ABHD6*, *ACAD9* and *PARVB*), immune response (*SKAP1*) and regulation of DNA transcription and RNA translation (*CDKAL1*, *E2F3*, *MXD1*, *RPP14*, *SNRNP27*, *SSBP2* and *ZXDC*).

*ABCA12* mediates lipid transporter activity, signals receptor binding, and has active trans-membrane transporter activity [50, 51]  identified that *ABCA12* is the major gene that influences Harlequin Ichthyosis in humans and later was identified in livestock species. *ABCA12* was associated with birth weight in Holstein cattle [52]. Monoacylglycerol lipase (*ABHD6*) is a lipase and the major enzyme for bone morphogenic protein catabolism, which plays a key role in the formation of intraluminal vesicles and in lipid sorting [53]. Although not previously associated with SC or fertility traits in cattle breeds, *ABHD6* was associated with average daily gain in crossbred beef cattle [54] as well as identified as a positional candidate gene for milk cholesterol in dairy cattle [55]. *ACAD9*, which catalyses the rate limiting step during the beta-oxidation of fatty acyl-CoA, was identified as a CNV in Italian sheep [56] and was found to be significantly associated with intramuscular fat content in Large White pigs [57]. Parvin beta (*PARVB*) is essential in establishing and/or maintaining cell polarity as well as actine cytoskeleton reorganization. Although not previously reported in beef cattle, a study on ketosis in German Holstein cattle [58] associated *PARVB* with non-alcoholic fatty liver disease, indicating that *PARVB* could be a part of lipid metabolism. An investigation into selection signatures in Indian swamp buffaloes identified *PARVB* [59].

Two genes previously reported to be linked to fertility traits in cattle include *FXN* and *GMCL1*. Frataxin (*FXN*) promotes the biosynthesis of heme and plays a role in the protection against iron-catalysed oxidative stress. *FXN* was the second ranking SNP in a logistic regression analysis of pregnancy status in Santa Gertrudis cows [60]. *GMCL1* appears to be located in an extended selection signature that shows high haplotypic homozygosity [61] and was previously associated with fertility traits in beef cattle breeds [62]. Single Stranded Binding Protein (*SSBP2*), located on BTA 7, is a candidate QTL for conferring resistance to Johne’s disease in cattle [63, 64]. *SSBP2* is involved in the positive regulation of transcription by RNA polymerase II and transcription by RNA polymerase II. A study in Holstein cattle associated *SSBP2* with body conformation traits [65]. *PPP3R1* is a regulatory subunit of calcineurin, which plays a role in neuronal calcium signalling. A study on transcriptome profiling of muscle in Nelore cattle [66] identified *PPP3R1* participates in mitogen-activated protein kinase (MAPK) signalling pathway which is responsible for cell proliferation, differentiation, and apoptosis.

A range of genes identified to be associated with SC have not been previously identified in cattle, but have been in water buffaloes and crossbred buffaloes. *ALDH1L1* [67], *TJP2* [67] and *TCOM5B* [68] were all linked to milk composition traits, more specifically fat and protein yield. The QTL containing *ALDH1L1*, *CHST13*, *SLC41A3*, *TXNRD3*, *UROC1* and *ZXDC* on BTA 22 was previously reported to be associated with somatic cell score in Holstein cattle [69]. The *TXNRD3* genes is known to affect adipocyte differentiation through the Wnt signalling pathway [70].

On BTA 19, an SC-associated QTL harbours four homeobox genes and *TTLL6*. *TTLL6* is a gene in a strong candidate region, which included *HOXB7* and *HOXB9*, for controlling skeletal tail length in sheep [71]. The four *HOXB* genes in this QTL have not been previously reported in beef cattle, but other *HOX* gene clusters are known to affect sperm quality in humans [72]. It is known that poor sperm DNA methylation is associated with decreased male fertility and low embryo quality. The hypomethylation of microRNA and *HOX* gene clusters play a significant role in embryonic development and is evidence of the sperm’s epigenetic contribution. *KIF2A* is known modulate mitotic events during spermatogenesis [73].

A limited number of studies have considered SC as a direct trait of interest for genomic investigations. Reviews of literature on bull fertility highlight the biological processes associated with sperm quality, motility, and scrotal volume [24, 74, 75]. More recent association studies revolve around sexual precocity, especially in tropical cattle [32, 74, 76–79] located in Central and Southern America.

### Overlapping genes across traits

Of the genes observed to be significantly associated with both AFC and ICP in the present study, *PLCB1* was identified to be linked to stay-ability in Nelore cattle [80]. *PLCB1* is known to be a target of a micro interfering RNA (*miR-301b*), which has been associated with ovarian follicle development in cattle breeds [81]. Puberty in a heifer occurs upon ovulation of a potentially fertile oocyte [44], while [82, 83] stress the importance of proper nutrition postpartum in order to re-establish ovarian activity for a shortened ICP. *PLCB1* is an enzyme that hydrolyses phospholipids into fatty acids as well as other lipophilic molecules and is involved in oxidative stress responses [84]. The regulation of adipose tissue affects the metabolic hormone leptin, known to regulate reproductive function in female animals [85, 86]. Twelve haplotype blocks for *PLCB1* were identified through an association analysis of carcass traits in Hanwoo cattle [42] and through ontology of this gene linked it to lipid metabolism. *ARAP3*, a GTPase-activating protein, and the multiple genes that are members of the *PCDH* family group have been reported as a selection signature in cattle related to immune response [87]. Although immunological studies in livestock species are limited, [88] reviewed the effect immune cells have on ovarian follicle development and the establishment of pregnancy.

*FCHSD1* and *RELL2* are located in a long run of homozygosity (ROH) detected in multiple Alpine-based dual-purpose breeds. These two genes are involved in the MAPK14/p38 cascade as well as apoptosis. Clathrin interactor 1 (*CLINT1*) plays a major role in the formation of coated vesicles. This gene was associated with milk yield, fat yield and percentage as well as protein yield and percentage in dairy cattle [84], while was linked to milk fat content in Simmental cows [43]. *LSM11* and *THG1L* have not been previously reported in cattle breeds but have been associated with milk protein yield and milk protein percentages in Valle del Belice dairy sheep [40]. *LSM11* being a small nuclear RNA that has processes the mRNA 3’-end prior to translation while *THG1L* is involved in the regulation of tRNA processing during translation.

The number of overlapping genes co-located in QTL shared by AFC and ICP, alongside the moderate genetic correlation (0.37) in this study, indicates fertility is initiated, regulated and maintained by pleiotropic genetic mechanisms. Multiple genes in this study have no obvious direct link with fertility traits and this further demonstrates the complexity of genetic mechanisms for traits such as AFC and ICP.

## Conclusion

In this study numerous genes, *ARAP3*, *CLINT1*, *FCHSD1*, *LSM11*, *PLCB1*, *RELL2*, *SM11* and *THG1L* were co-located in QTL that had a significant or suggestive association with both AFC and ICP. Numerous QTL were identified across 14 autosomes for SC, the majority of which had never been previously reported to be linked to reproductive traits. The identification of different genes with similar molecular and biological characteristics for these sex-limited traits reaffirms our understanding that these lowly heritable traits are influenced by many genes each contributing a small amount to the variation in these traits’ expression. Some genes related to carbohydrate catabolic processes, cellular development, iron homeostasis, lipid metabolism and storage, immune response, ovarian follicle development and the regulation of DNA transcription and RNA translation were identified as candidate genes for reproductive traits in SA Bonsmara cattle.

## Methods

### Genotypic data

Genotypes from 7,326 SA Bonsmara animals originating from one of five possible genotype arrays were available. A total of 1,950 animals were genotyped on the GeneSeek Genomic Profiler (GGP) 150K (140,113 SNPs), while 597 animals were genotyped on the GGP 80K (76,883 SNPs), 2,625 animals were genotyped on the Versa 50K (49,855 SNPs), 1,326 animals were genotyped on the SASB 50K (54,394 SNPs) with the remaining 828 on the ICBF IDB v.2 platform (52,445 SNPs). Only autosomal SNPs with a known base pair position, a call rate ≥ 0.90, a MAF ≥ 0.10 and did not significantly deviate from Hardy-Weinberg equilibrium (*p* > 0.001) were retained. All SNP locations were based on the UMD 3.1 genome build (GCF_000003055.6; [89]). Animals had a call rate of > 90%, while individuals with ≥ 0.95 identical genotypes were discarded as were families with more than 10% Mendelian errors. Quality control of SNP data was carried out using PLINK v.1.9 [90].

### Population stratification

Identical by state genetic distances between animals were computed through a MDS analysis with PLINK v.1.9 [90]. The analysis involved a total of 7,231 SA Bonsmara genotypes at a density of 24,216 SNPs that are truly genotyped across all five arrays. Visualisation of the data, reduced into two dimensions, allowed for the detection of possible population stratification as well as outliers. The remaining SA Bonsmara genotypes (4,403 males, 2,725 females) were imputed to 120,692 SNPs using FImpute v.3 [91].

### Phenotypic data

The SA Bonsmara minimum breed standards [92] indicate that a heifer must calve before 39 months of age and the first ICP cannot exceed 790 days. SA Bonsmara animals occur throughout all nine of South Africa’s provinces and are mainly raised in extensive natural pasture systems. The recording of weaning weight (205-day weight) is compulsory and facilitates the selection of bulls for post-weaning growth tests. Scrotal circumference is measured on bulls participating in central and farm-based growth tests at around 12 to 18 months of age. Standardised phenotypes for AFC (days), first ICP (days), and SC (millimetres) were available on 347,749 records for AFC, 206,505 for records ICP and 238,454 records for SC in individual SA Bonsmara animals from the LOGIX Genetic Evaluation System [93]. This was accompanied by pedigree information on 2,135,235 animals dating back to 01 June 1949, as well as data on the contributing systematic environmental effects associated with these traits.

### Deregression of breeding values

In order to predict estimated breeding values, a bivariate animal linear model for AFC and ICP and a univariate animal linear model for SC were defined as follows;

$$\varvec{y} = \varvec{Xb} + \varvec{Zu} + \varvec{e}$$

where,

**y** is the vector of phenotypes for AFC, ICP and SC;

**b** is a vector of fixed effects which include sex, herd, birth month and year, age in days at measurement of the phenotype covariate (linear regression);

**u** is a vector representing the direct additive-genetic effects, with **u** ~ N(0,A ${\sigma }_{u}^{2}$), where A is the pedigree-based matrix and ${\sigma }_{u}^{2}$ is the direct genetic variance;

**e** represents the residual, where e ~ N(0,**I**${\sigma }_{e}^{2}$), with ${\sigma }_{e}^{2}$ representing the residual variance and **I** the identity matrix;

**X** and **Z** are incidence matrices for **b** and **u** respectively.

Estimation of variance components for the animal model stated above was calculated using restricted estimated maximised likelihood (REML) optimised with quasi-Newton procedure using analytical gradients in Variance Component Estimation (VCE) [94] software. MiX99 [95] was used to predict breeding values for AFC, ICP and SC using the same model in the estimation of variance components. Effective record contributions (ERCs) for each animal and trait were generated as described in [96] using the reversed reliability approximation method in APaX99 [97]. The EBVs of the genotyped animals for each trait were then deregressed using the Secant method [98] in MiX99 [95] alongside the generated ERC. Deregressed EBVs (DEBVs) were weighted using the formula set out by [99];

$${{w}}_{i}=\frac{1-{{h}}^{2}}{\left[{c}+\frac{1-{{r}}_{i}^{2}}{{{r}}_{i}^{2}}\right]{{h}}^{2}}$$

where,

***w*** is the weighting factor of the *i*th animal with a DEBV;

***h***^***2***^ is the heritability estimate for the respective traits,

***r***^***2***^ is the reliability of the DEBV for the *i*th animal for a specific trait and,

***c*** is the proportion of genetic variance not accounted by the SNPs with a value of 0.90 being used for all weighting factors between all the traits under analysis.

Only animals with an ERC ≥ 0.50 and a reliability ≥ 0.01 were retained for each trait analysis.

### Association analyses

A genomic relationship matrix (GRM) was constructed for each trait using the VanRaden method 1 [100]. Additive and residual genetic variances for each trait were computed via genomic REML (GREML) using GCTA v1.94 [101]. Weighted DEBVs were regressed on each SNP individually using a linear mixed model in WOMBAT [102].

$$\varvec{y} = \varvec{\mu} + \varvec{SNP} + \varvec{a} + \varvec{e}$$

where,

**y** is the vector of phenotypes, the weighted DEBV;

**µ** is the fixed effect of the population mean;

**SNP** is the fixed effect of allele dosage for each SNP (coded as 0, 1 or 2);

**a** is the random effect of the animal, where a ~ (0,${\varvec{G}\sigma }_{a}^{2}$), with ${\sigma }_{a}^{2}$ representing the additive genetic variance of the animal;

**G** is the genomic relationship matrix among animals,

**e** represents the residual, where e ~ N(0,**I**${\sigma }_{e}^{2}$),

with ${\sigma }_{e}^{2}$ representing the residual variance and **I** the identity matrix.

The t-test statistics for all SNPs were obtained and subsequently transformed into lower tail p-values. To minimise false positives, the Benjamini-Hochberg False Discovery Rate (B-H FDR) method was applied to each SNP. SNPs with a *P* ≤ 4 × 10^− 7^ were considered to be genome-wide significant as per Bonferroni correction, with SNPs with a *P* ≤ 4 × 10^− 6^ being deemed suggestive. Manhattan plots, Figs. 3, 4 and 5, were generated in R using the qqman [103] package.

### Defining QTLs and candidate genes

The extent of LD among significant SNPs (*P* ≤ 4 × 10^− 7^) was estimated, as was the pairwise LD among all SNPs within 5 Mb up and downstream of the significant SNP [104]. The start and end of each QTL was defined by SNPs furthest up and downstream of the significant SNP and had an r^2^ > 0.50 with other significant SNPs. If any QTL were deemed to be overlapping, these were consolidated into one large QTL. If no SNPs were in LD with the significant SNP, that SNP was deemed a quantitative trait nucleotide. Identified QTL were then explored using ENSEMBL (https://www.ensembl.org/) according to the UMD 3.1 genome build in order to detect candidate genes residing within and Panther [105] was used to list the biological and metabolic functions and/or processes of possible genes.

**Table 3:** List of identified QTL and genes significantly associated with Scrotal Circumference

BTA^1^	SNPs in QTL^2^	Start - End	Significant SNP	P-value	MAF^3^	Genes in quantitative trait loci
2	6	103647078–103740281	ARS-BFGL-BAC-5705	2.96E-07	0.4904	*ABCA12, RF00156 and a CNV*
2	1	106952479–106952479	BTA-48880-no-rs	2.04E-07	0.4164	
3	1	78383390–78383390	ARS-BFGL-NGS-67919	2.01E-07	0.1769	
5	1	94407955–94407955	BovineHD0500026808	2.36E-07	0.1589	*DERA*
5	7	115473272–115577302	ARS-BFGL-NGS-38686	2.04E-07	0.1882	*PARVB*
7	2	83800568–83815912	BovineHD0700024601	5.60E-08	0.1386	*SSBP2*
8	5	45501201–45696078	BovineHD0800013600	8.38E-08	0.1396	*FXN, TJP2 and 3 CNVs*
10	1	29117018–29117018	BTB-00415713	6.67E-09	0.0643	
10	1	29126891–29126891	BovineHD1000009600	3.10E-10	0.3962	*TMCO5B*
10	1	29621558–29621558	BovineHD1000009772	2.37E-07	0.2387	
10	4	32006964–32221643	Hapmap30523-BTA-133067	3.37E-07	0.1802	*2 CNVs*
11	3	66622010–66705064	BovineHD1100018839	2.05E-07	0.4144	*PPP3R1, CNRIP1*
11	3	67319888–67350067	BovineHD1100018999	2.24E-07	0.226	*ANTXR1*
11	2	67577210–67626052	ARS-BFGL-NGS-32754	2.01E-07	0.3291	*ANTRX1*
11	2	67847635–67869723	BovineHD1100019186	2.01E-07	0.3463	*AAK1*
11	2	68190789–68274343	BovineHD1100019275	2.01E-07	0.0394	*GMCL1, MXD1, SNRNP27*
12	5	60509480–60612829	ARS-BFGL-NGS-61380	8.38E-08	0.4923	
14	5	78092917–78209050	BovineHD1400021894	3.09E-07	0.2385	*CNV*
19	1	32819208–32819208	BovineHD1900009676	2.05E-07	0.3072	
19	1	37784677–37784677	BovineHD1900010983	3.68E-10	0.2839	
19	1	38242204–38242204	BovineHD1900011094	3.68E-10	0.3803	*UBE2Z*
19	3	38370506–38542284	BovineHD1900011158	3.68E-10	0.4151	*HOXB5, HOXB7, HOXB9,HOXB513, TTLL6*
19	1	38912461–38912461	BovineHD1900011204	3.20E-10	0.2616	*SKAP1*
19	1	39183738–39183738	BovineHD1900011267	3.68E-10	0.0205	*PNPO*
19	1	52841109–52841109	BovineHD1900014798	1.58E-08	0.3857	*CNV*
19	1	53473744–53473744	ARS-BFGL-NGS-103691	2.96E-07	0.279	
19	1	62932571–62932571	BovineHD1900018179	3.63E-07	0.3632	*CNV*
20	11	4356597–4395656	BovineHD2000001405	5.77E-09	0.1642	*NEURL1B*
20	1	16209098–16209098	BovineHD2000004865	3.20E-10	0.1877	
20	2	17075769–17124999	BovineHD2000005141	2.16E-08	0.133	*KIF2A*
20	1	20720490–20720490	BovineHD2000006199	1.31E-09	0.357	*RAB3C*
20	1	31848979–31848979	ARS-BFGL-NGS-10108	2.70E-07	0.4553	
22	3	43503370–43595820	Hapmap33950-BES3_Contig483_1359	4.02E-09	0.4245	*ABHD6, PXK, RPP14*
22	2	45589797–45681461	BovineHD2200013184	2.04E-07	0.4508	*RF00100*
22	2	59576240–59596062	BovineHD2200017302	2.18E-08	0.369	*ACAD9*
22	5	61086693–61224609	ARS-BFGL-NGS-105794	3.61E-07	0.1034	*ALDH1L1, CHSR13, TXNRD3, UROC1, ZXDC*
23	1	22245990–22245990	BovineHD2300005886	3.20E-10	0.0961	*CRISP1*
23	1	22536307–22536307	BovineHD2300005950	1.94E-07	0.3384	
23	1	31768035–31768035	BovineHD2300009170	5.02E-11	0.0391	*SLC17A3*
23	6	37029269–37422153	BovineHD2300010762	8.53E-10	0.4092	*CDKAL1, E2F3*
29	6	13935802–14009294	BovineHD2900004129	2.04E-07	0.255	

^1^*BTA Bos taurus* autosome.

^2^***SNPs*** *in QTL* Number of single nucleotide polymorphisms in this quantitative trait loci.

^3^MAF Minor allele frequency of the significant single nucleotide.

## Data Availability

The datasets generated and/or analysed during the current study are available at 10.6084/m9.figshare.21800117.

## Abbreviations

AFC: Age at first calving
BGP: Beef Genomics Program
BTA: *Bos taurus* autosome
EBV: Estimated breeding value
DEBV: Deregressed estimated breeding value
ERC: Effective record contribution
GGP: Geneseek genomic profiler
GRM: Genomic relationship matrix
GWAS: Genome-wide association studies
h^2^: Heritability
IBS: Identical by state
ICP: Inter-calving period
LD: Linkage disequilibrium
MAF: Minor allele frequency
MDS: Multidimensional scaling
QTL: Quantitative trait loci
REML: Restricted estimated maximum likelihood
ROH: Run of homozygosity
SA: South Africa
SASB: South African Stud Book
SC: Scrotal circumference
SE: Standard error
SNP: Single nucleotide polymorphism
